# Supplementary material for: Re-evaluation of neuronal P2X7 expression using novel mouse models and a P2X7-specific nanobody
Source: eLife. 2018 Aug 3;7:e36217. doi: 10.7554/eLife.36217 (PMC6140716; doi:10.7554/eLife.36217)
Supplement: Supplementary file 1. [file elife-36217-supp1.docx]

**Supplementary file 1–Probes**

| Probe | Name | Sequence |
| --- | --- | --- |
| StrepHisEGFP insertion  Hom. recomb. 1  (GalK insertion)  Hom. recomb. 2  StrepHisEGFP insertion  L451P exchange  Hom. recomb. 1  (GalK insertion)  Hom. recomb. 2  L451P exchange | X7Ex13GalKfw  X7Ex13GalKrev  HomAEx13X7GFP  HomBEx13X7GFP  X7L451PgalK_F  X7L451PgalK_R  X7L451P_S  X7L451P_AS | GAAGGAGTTCCCCAAGACCGAGGGGCAGTATAGTGGCTTCAAGTATCCCTACCCTGTTGACAATTAATCATCGGCA  CTAGGTCTTTCCAAGGGAAGCTGTATTGTGAGCCACCATGATGTGGCAGCCGTACCATCATCAGCACTGTCCTGCTCCTT  GAAGGAGTTCCCCAAGACCGAGGGGCAGTATAGTGGCTTCAAGTATCCCTACAGCGCCTGGAGCCACCCGCAGTTC  CTAGGTCTTTCCAAGGGAAGCTGTATTGTGAGCCACCATGATGTGGCAGCCGTACCATCATTACTTGTACAGCTCGTCCATG  GGACTTCTCCGACCTGTCTAGGCTGTCCCTATCTCTCCACGACTCACCCCCCTGTTGACAATTAATCATCGGCA  GGGCCACCTCTTCATGGAGCAGCTGAATTTCCTCAGATTGTCCAGGAGTCTCAGCACTGTCCTGCTCCTT  GGACTTCTCCGACCTGTCTAGGCTGTCCCTATCTCTCCACGACTCACCCCCGACTCCTGGACAATCTGAGGAAATTCAGCTGCTCCATGAAGAGGTGGCC  GGCCACCTCTTCATGGAGCAGCTGAATTTCCTCAGATTGTCCAGGAGTCGGGGGTGAGTCGTGGAGAGATAGGGACAGCCTAGACAGGTCGGAGAAGTCC |
| X7intr/ex13 probe (606 bp)  EGFP probe  (604 bp)  Southern blot  probes | Forward primer  Backward primer  Forward primer  Backward primer Forward primer  Backward primer | gtgctgggattaaaggtgtgc AGCTTATGGAAGAGCTTGGAGGT  GTAAACGGCCACAAGTTCAGC  ACTCCAGCAGGACCATGTGAT GCTTGTGATAAGGACGCC  TTTTTGGTCTACTGCGTG |
