# Supplementary material for: Re-evaluation of neuronal P2X7 expression using novel mouse models and a P2X7-specific nanobody
Source: eLife. 2018 Aug 3;7:e36217. doi: 10.7554/eLife.36217 (PMC6140716; doi:10.7554/eLife.36217)
Supplement: Supplementary file 2. [file elife-36217-supp2.docx]

**Supplementary file 2–Primers**

| Primer for | Name | Sequence |
| --- | --- | --- |
| Genotyping | seqX7Ex13_F  (in intron 12)  X7BAC5_R  (in EGFP)  or  seqX73UTR_R  (in 3´UTR) | GGTTCTTAGCAGGCTTAACAGCA  ATGGGGGTGTTCTGCTGGTAGT  GCCATTGGTCTAATCAGCTCTC |
| Real time PCR | TM_Pdhb_for  TM_Pdhb_rev  (House keeper,  Roche probe #4)  TM_P2X7_for  TM_P2X7_rev  (Roche probe #42)  TM_Iba1_for  TM_Iba1_rev  (Roche probe #67)  TM_ Tnfa_for  TM_ Tnfa_rev  (Roche probe #68)  TM_ Il1b_for  TM_ Il1b_rev  (Roche probe #38) | TTAAATCGGCCATTCGTGAT  CAGGAAATCTTTTGACTGAGCTT  CTGGTTTTCGGCACTGGA  CCAAAGTAGGACAGGGTGGA  ATCTGCCGTCCAAACTTGA  CTAGGTGGGTCTTGGGAACC  CTGTAGCCCACGTCGTAGC  TTTGAGATCCATGCCGTTG  AGTTGACGGACCCCAAAAG  AGCTGGATGCTCTCATCAGG |
